# Artificial Intelligence-Enabled Intelligent Sensory Systems for Quality Evaluation of Traditional Chinese Medicine: A Review of Electronic Nose, Electronic Tongue, and Machine Vision Approaches

**DOI:** 10.3390/molecules31071140

**Published:** 2026-03-30

**Authors:** Jingqiu Shi, Jinyi Wu, Li Xu, Ce Tang, Yi Zhang

**Affiliations:** 1 Chinese Medicine Germplasm Resources Innovation and Effective Uses Key Laboratory of Sichuan Province, School of Pharmacy, Chengdu University of Traditional Chinese Medicine, Chengdu 611137, China; shijingqiu@stu.cdutcm.edu.cn (J.S.); 18982066975@163.com (J.W.); 2 Chengdu Institute for Drug Control, NMPA Center for Innovation and Research in Regulatory Science, Chengdu 610045, China; 3 Chinese Medicine Germplasm Resources Innovation and Effective Uses Key Laboratory of Sichuan Province, School of Ethnic Medicine, Chengdu University of Traditional Chinese Medicine, Chengdu 611137, China

**Keywords:** traditional Chinese medicine, intelligent sensory evaluation, artificial intelligence, data fusion, quality assessment

## Abstract

Traditional sensory evaluation of traditional Chinese medicine (TCM) and medicinal and food homologous products has long relied on human observation of appearance, color, aroma, and taste. However, this approach is highly subjective, difficult to quantify, and often lacks reproducibility across evaluators. Intelligent sensory systems, including the electronic nose, electronic tongue, and machine vision, provide objective and digitized sensory information for TCM quality evaluation. Nevertheless, these platforms generate high-dimensional and heterogeneous datasets, creating a strong demand for efficient artificial intelligence (AI)-based analytical tools. This review summarizes recent advances in the application of machine learning and deep learning methods, such as support vector machine, random forest, convolutional neural network, and long short-term memory networks, for intelligent sensory evaluation of TCM. Particular emphasis is placed on how AI supports feature extraction, pattern recognition, classification, regression, and multisource data fusion across electronic nose, electronic tongue, and machine vision systems. Representative applications in raw material authentication, geographical origin discrimination, processing monitoring, and quality grading are also discussed. In addition, the current challenges related to data standardization, sensor drift, model robustness, and interpretability are highlighted. Overall, this review provides an integrated overview of AI-enabled intelligent sensory technologies and clarifies their potential to advance TCM quality evaluation toward a more objective, efficient, and holistic framework.

## 1. Introduction

Traditional Chinese Medicine (TCM) is a unique medical system with demonstrated efficacy in disease prevention, treatment, and health maintenance [1,2,3]. Medicinal and Food Homology (MFH) products, which provide both nutritional and pharmacological benefits, are included in this review because their quality, like other TCM products, is influenced by complex factors such as botanical origin, geographical location, harvesting season, processing techniques, and storage conditions [4,5,6,7,8]. Traditional sensory evaluation, established since the time of Shen Nong, historically guided quality assessment based on appearance, aroma, taste, and other sensory attributes [9]. Although culturally significant, these methods are subjective, difficult to quantify, and limited in reproducibility [10,11]. Modern analytical techniques, such as chromatography and mass spectrometry, have enhanced the precision of TCM quality evaluation by providing detailed chemical composition data. However, challenges remain, including the complexity of TCM matrices, variability in raw materials, and limited reproducibility across laboratories [12,13].

To overcome the limitations of traditional sensory evaluation, intelligent sensory technologies have been developed to simulate human sensory perception and transform sensory characteristics into objective digital signals. These technologies mainly include electronic noses (E-nose), electronic tongues (E-tongue), and electronic eyes (E-eye). The E-nose detects volatile compounds, the E-tongue quantifies taste-related attributes, and the E-eye captures visual features such as color and morphology. Together, these systems enable the systematic characterization of the shape, color, aroma, and taste of medicinal materials. Initially developed for food analysis [14], intelligent sensory technologies have rapidly expanded into pharmaceutical analysis [15], medical diagnostics [16], and environmental monitoring [17], demonstrating increasing potential for TCM quality evaluation.

At the same time, these sensing systems generate complex, high-dimensional datasets that often contain nonlinear relationships, redundancy, and noise, making conventional linear statistical analysis insufficient in many cases [18,19]. For example, one study reported a 72-channel MOS sensor array responding to ten gaseous substances [20], while machine vision images may contain millions of pixels encoding texture and color information [21]. In this context, artificial intelligence (AI), particularly machine learning (ML) and deep learning (DL), has become an essential analytical tool for extracting meaningful patterns from intelligent sensory data and linking them to quality-related attributes such as origin, authenticity, processing degree, and pharmacological properties [22,23].

This review focuses on one of the most promising directions in this field: the integrated application of AI and multisource intelligent sensory technologies for TCM quality evaluation. It systematically discusses electronic nose, electronic tongue, and electronic eye systems together with representative machine learning and deep learning algorithms, multisource information fusion strategies, and their major applications in TCM and MFH quality control. The ultimate goal is to support the development of a more intelligent, objective, and holistic framework for next-generation TCM quality evaluation.

The literature discussed in this review was retrieved mainly from PubMed, Web of Science, Scopus, and CNKI. The search focused on combinations of keywords such as “traditional Chinese medicine”, “medicinal and food homologous products”, “electronic nose”, “electronic tongue”, “machine vision”, “artificial intelligence”, “machine learning”, “deep learning”, and “data fusion”. Priority was given to studies published in recent years that were directly related to the quality evaluation of TCM and MFH, especially those involving methodological development, representative applications, multisource fusion, and current challenges. Studies with limited relevance to intelligent sensory evaluation or those lacking clear methodological information were not emphasized in the discussion.

Compared with previous reviews that often focus on individual sensing technologies or isolated analytical methods, the present review provides a more integrated perspective by jointly examining electronic nose, electronic tongue, and electronic eye systems in combination with AI algorithms, multisource fusion strategies, representative quality evaluation applications, and the major challenges and future directions of this field. In this way, this review aims not only to summarize current methodological progress, but also to highlight the broader significance of integrating intelligent sensory technologies with AI in advancing a more objective and holistic framework for TCM quality evaluation.

## 2. Intelligent Sensory System

### 2.1. Basic Principles

The core of intelligent sensory technology lies in the simulation of human sensory perception. Although intelligent sensory devices are designed for different sample types, they share common features in their fundamental architecture and operating principles. E-noses and E-tongues serve as representative examples and are generally composed of three core components: a sensor array, a signal acquisition unit, and a computing system. The sensor array mimics human olfactory or gustatory receptors by interacting with samples and generating corresponding response signals. Analogous to the peripheral nervous system, the signal acquisition unit is responsible for signal transmission and preliminary preprocessing, such as amplification and filtering. The preprocessed signals are then fed into the computing system, which corresponds to the central nervous system. Here, a general data analysis workflow is implemented: first, relevant features are extracted from the signals; subsequently, machine learning algorithms—such as principal component analysis (PCA), support vector machines (SVMs), or neural networks—are applied for dimensionality reduction, pattern recognition, and classification. This process ultimately generates a comprehensive profile of the sample, enabling accurate identification or quality assessment [24,25].

E-eyes, commonly referred to as machine vision (MV) systems, differ substantially in structure from electronic noses and electronic tongues. Essentially, E-eye systems are computer vision platforms consisting of a digital camera, image processing software, and supporting mechanical structures. A dedicated lighting system provides controlled and uniform illumination, enabling high-fidelity image acquisition. The acquired images are processed by specialized software, and the resulting analytical outputs directly guide the mechanical subsystem to execute targeted actions [26].

Olfaction, one of the essential human senses, plays a crucial role in environmental perception and adaptive survival mechanisms. Advances in sensing technologies have fueled interest in simulating biological olfaction, leading to the development of key devices such as the electronic nose. The electronic nose (E-nose) detects volatile compounds via an array of chemical sensors that respond selectively to different odorants. Interactions at the sensor surfaces generate electrical signals proportional to the concentrations of target compounds. These signals are then preprocessed and converted into feature vectors, which are analyzed using pattern recognition algorithms to classify or quantify the odor profiles [27].

Similar to the electronic nose, an E-tongue is an analytical instrument that simulates human taste perception, allowing for the quantitative assessment of various compounds’ taste attributes. It typically consists of a working electrode and a reference electrode, which measure changes in inter-electrode resistance or generated current signals to characterize the sample’s taste profile [28]. The acquired analog signals are digitized and fed into mathematical models, where they are processed by a computer system to simulate and evaluate the perceived taste features [29].

MV is a technological system designed to simulate human visual functions. In image recognition tasks, machine vision systems analyze image content by classifying objects and assigning categorical labels, enabling the identification of specific items, distinguishing features, or broader scene contexts [30]. The system operates in three key stages: image acquisition, which acts as the system’s “eyes” by converting optical information into digital signals; image processing and analysis, functioning as the visual cortex to preprocess these signals and extract features using techniques such as mean filtering, Gaussian filtering, and wavelet denoising to enhance relevant information and suppress noise [31]; and image recognition, which serves as the brain, where algorithmic models compare and evaluate the extracted features. Although traditional ML methods, such as SVMs, Decision Trees (DTs), and K-Nearest Neighbors (KNNs), were once widely used, deep learning (DL) approaches have now taken the forefront in research. Methods such as Artificial Neural Networks (ANNs) and Convolutional Neural Networks (CNNs) have significantly enhanced recognition performance and overall intelligence in machine vision systems.

Although intelligent sensory systems provide a promising route for objective and digitized quality evaluation, their practical application is still constrained by several limitations. First, sensor stability remains a concern, since long-term use, environmental fluctuations, and sensor aging may lead to signal drift. Second, reproducibility is not always satisfactory, particularly when different devices, sensor arrays, or sample preparation procedures are used across laboratories. Third, reliable performance often depends on regular calibration and standardized preprocessing workflows. Therefore, further improvements in sensor robustness, inter-platform consistency, and calibration strategies are still needed for broader application of these systems in TCM quality evaluation [32].

### 2.2. Historical Development

**Figure 1:**
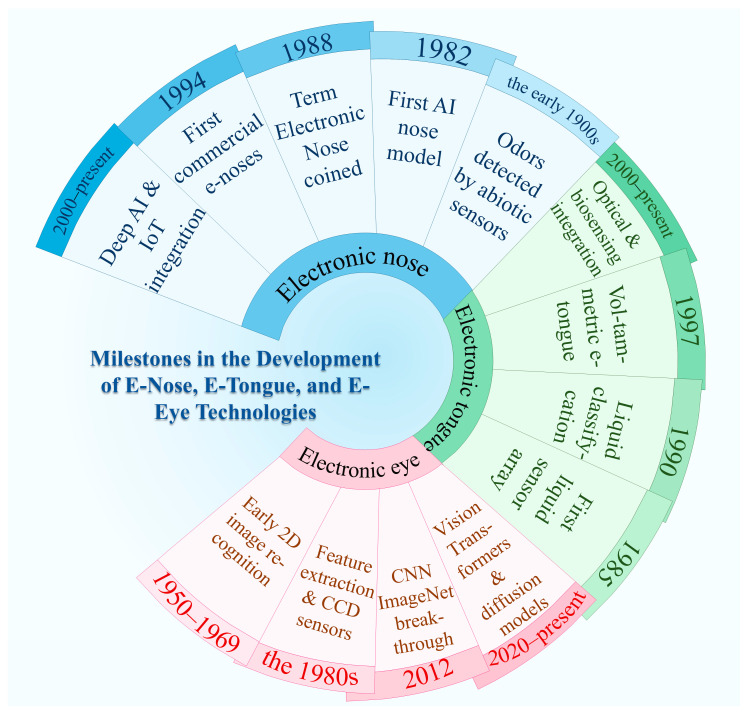
Historical review and key milestones in development of intelligent sensory systems.

The evolution of intelligent sensory instruments has transitioned from single-function devices to integrated systems, moving from an auxiliary role to that of sensory replacements. In the 1970s, researchers at Bell Laboratories in the United States successfully developed the charge-coupled device (CCD) sensor. This hardware breakthrough enabled the first efficient and high-fidelity conversion of optical images into digital signals, laying the technological foundation for the digitization of visual information [33]. This was followed by the feasibility of quantitative perception of smell and taste. In 1982, Persaud and Dodd at the University of Warwick developed the first E-nose prototype, which was based on a sensor array and pattern recognition system inspired directly by mammalian olfaction [34]. In the 1990s, Professor Toko’s team in Japan achieved the qualitative identification of liquid samples, a milestone widely considered the first true taste sensor [35]. A central theme of this evolution is the conversion of subjective sensory perceptions into quantifiable, objective data. However, the widespread adoption of these instruments has led to the generation of high-dimensional, large datasets that exceed the capabilities of traditional analytical methods. This challenge has made the integration of artificial intelligence not only beneficial but essential, ushering in a data-driven era that requires intelligent systems to decode complex sensory information. Figure 1 illustrates the historical development trajectory.

### 2.3. Exploration and Validation of Evaluation of Properties and Flavors of TCM

The greatest value of intelligent sensory technology in TCM quality control lies in its ability to provide quantifiable scientific tools for assessing the principles of *Bian Zhuang Lun Zhi* (evaluating quality by appearance), medicinal properties, flavor, and meridian tropism, thus bridging traditional experience with modern data. By converting the macroscopic characteristics of herbs into digital signals, researchers can systematically develop correlation models that link traditional empirical features with modern chemical data. The inherent energy of Chinese herbs, known as Qi, while not fully synonymous with the four properties of medicinal nature, shows a strong correlation. For example, aromatic properties often correspond to the nature of invigorating the spleen, transforming dampness, and dispersing. Research indicates that the response of the electronic nose sensor to *Atractylodis Macrocephalae* Rhizoma strongly correlates with the content of its primary active ingredient, atractylenone [36]. Atractylenone, a key component of its volatile oil, interacts synergistically with other volatile constituents, forming the material basis for the aromatically transforming dampness efficacy of *Atractylodes lancea* (Thunb.) DC. [37]. Therefore, the data captured by electronic noses reveal the chemical patterns underlying TCM properties, offering a modern approach for objectively evaluating medicinal properties from the olfactory dimension.

Taste quantification primarily depends on electronic tongue technology. The theory of the five flavors in TCM highlights the close relationship between taste and therapeutic efficacy, exemplified by axioms such as bitterness clears heat and sweetness tonifies deficiency [38]. By converting taste sensations into intensity data through sensor arrays, electronic tongues facilitate the development of mathematical models that connect digital taste profiles to meridian tropism and efficacy. For instance, a study on the bitter compounds of *Platycodon grandiflorum* (Jacq.) A. DC demonstrated a strong correlation between its bitterness and several saponin components [39]. These saponins specifically bind to the human bitter taste receptor TAS2R14, and their documented antitussive, expectorant [40], and anti-inflammatory [41] effects provide a modern counterpart to the traditional axiom that “bitterness drains fire”. Similarly, research on *Ligusticum chuanxiong* Hort. shows that its pungency, captured by the electronic tongue, primarily originates from volatile oil components [42]. Modern pharmacology confirms that these components induce vasodilation, improve microcirculation, inhibit platelet aggregation, and exhibit anti-inflammatory and analgesic effects, a profile that aligns precisely with the TCM principle that pungency promotes movement and dispersion. Therefore, the pungency quantified by the electronic tongue serves as a digital representation of Chuanxiong’s traditional efficacy in promoting blood circulation, activating Qi, dispelling wind, and alleviating pain.

The electronic eye system is responsible for digitally deconstructing the morphology and color of medicinal materials. The external characteristics of medicinal materials provide critical evidence for evaluating quality based on appearance, reflecting their origin, growth duration, and processing techniques. For example, during the steaming process of *Gastrodia elata*, the electronic eye system objectively recorded color changes in the samples over extended steaming periods, including reduced brightness, deepening reddish-yellow hues, and increased saturation [43]. This color trajectory strongly correlated with changes in the concentrations of five major active components in *Gastrodia elata*. This demonstrates that the electronic eye detects not only superficial changes but also the external manifestation of internal chemical transformations.

In summary, intelligent sensory technology systematically aligns measurement dimensions, such as Qi, aroma, and appearance, with the core elements of TCM theory. This integration is driving the evaluation of TCM quality from an art based on individual experience to a science grounded in objective data. This transformation establishes a solid foundation for the development of a new intelligent evaluation paradigm that integrates traditional wisdom with modern technology.

### 2.4. Cutting-Edge Development Trends in Intelligent Sensory Technology

The frontier of electronic nose technology has advanced beyond conventional chemical sensor arrays through several important developments. In terms of sensitivity, bioelectronic noses incorporate brain–computer interfaces to record neural signals from the olfactory bulb of living organisms. These signals can then be decoded to enable ultra-trace gas detection at parts-per-billion concentrations [44]. In terms of energy efficiency, neuromorphic electronic noses mimic the brain’s spiking neural networks (SNNs), creating ultra-low-power sensing–computing architectures suitable for real-time and continuous monitoring [45]. In terms of specificity, material–algorithm co-optimization strategies have enabled highly selective detection of target components in complex mixtures using only a few sensors. Through the design of sensitive materials and feature engineering methods such as MACO, these systems can also improve model interpretability [46]. At the integration level, bionic olfactory chips fabricated through advanced micro/nano processes can integrate tens of thousands of sensing units onto a single chip, providing a hardware foundation for high-throughput and parallel odor information acquisition [47].

Although these technologies have not yet been widely applied in TCM and medicinal and food homologous product analysis, they represent a promising next generation of tools. In particular, they may help address practical challenges such as trace component detection, rapid on-site quality control, and more precise authentication of geoherbal materials.

The development of electronic tongue technology has also progressed beyond the simulation of basic tastes such as sourness, sweetness, bitterness, and saltiness. This shift has been driven by the integration of biosensing, microfluidic chip technology, and multidimensional signal analysis. For example, researchers have developed a reusable e-tongue that couples the capsaicin receptor (transient receptor potential vanilloid 1, TRPV1) with a carbon nanotube field-effect transistor [48]. This bioreceptor–nanomaterial strategy provides a promising prototype for mapping and quantifying medicinal sensory properties, such as pungency, in TCM. In addition, an MXene-based sensor integrated with microfluidic channels and modified with a Nafion membrane has achieved rapid and selective detection of H^+^ ions and has been applied to monitor pH dynamics during kimchi fermentation [48,49]. Such advances provide a feasible path toward miniaturized electronic tongues for real-time monitoring of key chemical changes during TCM processing or fermentation.

Electronic eye technology has likewise moved beyond conventional two-dimensional color imaging toward richer forms of multidimensional visual acquisition. Hyperspectral imaging (HSI), for instance, captures continuous spectral information across hundreds of wavelength bands for each pixel in a sample, enabling non-destructive visualization and quantitative assessment of internal chemical composition [50]. This creates new opportunities for evaluating the spatial distribution of active compounds in TCM and for supporting more precise quality grading and authenticity verification. Moreover, the integration of three-dimensional morphology, multispectral imaging, and thermal imaging has expanded the scope of visual sensing in biological materials [51]. These approaches may support early detection of pests, diseases, and mold in medicinal herbs, thereby improving pre-harvest quality monitoring. In addition, time-series approaches have enabled dynamic process monitoring, including the tracking of key phenological stages and trend prediction in crop development [52], further extending the role of electronic eye systems in monitoring biological processes.

Overall, intelligent sensory technologies are evolving toward higher specificity, greater throughput, miniaturization, and more comprehensive multidimensional sensing. For TCM research, these developments may provide stronger technical support for quality standardization, process monitoring, and authenticity assessment.

## 3. Smart Sensory Instruments: AI’s Data Factory

### 3.1. E-Nose and E-Tongue

Electronic noses and tongues operate on a similar principle: both use bionically designed sensor arrays to detect chemical stimuli. Their output is not a simple scalar value or image, but a temporally rich dataset, often represented as response curves or fingerprint spectra [53]. In E-nose systems, for example, volatile organic compounds are converted into electrical signals by a chemical sensor array [54,55]. This array typically consists of multiple cross-sensitive sensors, each responding differently to target analytes and collectively forming a composite spectral signature for the sample [56]. The raw signals are then preprocessed, relevant features are extracted, and dimensionality reduction may be applied to generate feature vectors suitable for subsequent modeling [57]. Such data are typically high-dimensional, dynamic, and nonlinear, which places clear demands on the choice of analytical methods.

Accordingly, both traditional machine learning and deep learning have been applied to E-nose and E-tongue data analysis. Traditional methods such as SVM and RF usually rely on manually constructed feature vectors and have shown stable performance in classification and regression tasks [32,58,59,60]. By contrast, deep learning models, including CNNs, RNNs, and LSTMs, are increasingly used to analyze sequential sensory signals and capture more complex patterns [61,62,63,64]. For example, ref. [61] proposed a Sensor-Aware Convolutional Network (SACNet) integrated with E-nose technology, achieving over 95% accuracy in classifying chili pepper varieties and tracing their geographical origin. Nevertheless, this result was obtained under a study-specific experimental setting, and its broader robustness across more heterogeneous samples and independent validation scenarios remains to be further established.

### 3.2. Electronic Eye

The E-eye system integrates optical imaging with image processing to provide a digital framework for the analysis of TCM materials. It captures external visual information, such as morphology, color, and texture, through image sensors including CCD cameras. For internal characterization, techniques such as magnetic resonance imaging (MRI) and X-ray imaging can also be introduced to reveal microscopic structures and internal composition [30].

**Figure 2:**
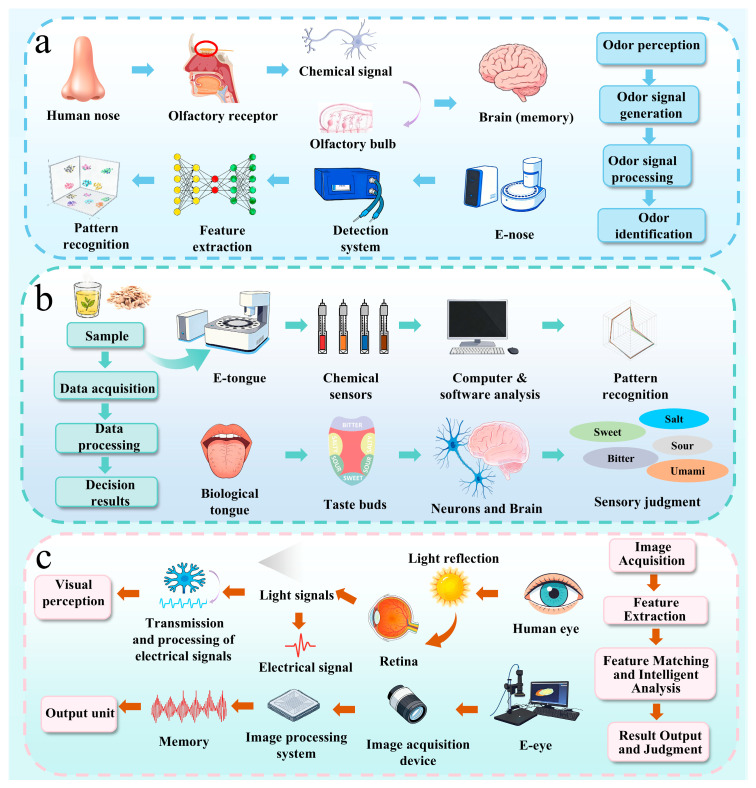
Working principles and key components of intelligent sensory instruments. (**a**) The electronic nose (E-nose) mimics the human olfactory system by detecting volatile compounds through sensor arrays, converting them into chemical signals, and performing feature extraction and pattern recognition for odor identification. (**b**) The electronic tongue (E-tongue) simulates taste perception by acquiring signals from chemical sensors, followed by data processing and pattern recognition to generate sensory judgments such as sweet, sour, bitter, salty, and umami. (**c**) The electronic eye (E-eye) imitates visual perception by capturing reflected light signals through image acquisition devices, followed by image processing, feature extraction, intelligent analysis, and final output of visual evaluation results.

After image acquisition, preprocessing steps such as size normalization, noise filtering, image enhancement, and region segmentation are applied to improve image quality and support subsequent recognition [65,66]. Feature extraction is a key step in this workflow. Traditional approaches usually rely on manually designed descriptors, whereas current image recognition methods increasingly adopt data-driven models to learn representative features directly from images. A schematic of the electronic eye working mechanism is shown in Figure 2c.

In a study on olive variety classification [67], the authors collected images of 2800 fruit samples representing seven varieties. After preprocessing, the images were used to train six different CNN architectures, among which Inception-ResNetV2 achieved the best performance, with an accuracy of 95.91%. Still, this result was obtained on a relatively well-defined image dataset, and its transferability to more heterogeneous medicinal materials or real-world grading scenarios remains to be further verified. In TCM research, E-eye systems have been applied to visual analysis of Chinese medicinal materials, including monitoring appearance changes during processing and appearance-based quality evaluation [43,68].

### 3.3. Multisource Information Fusion

Although single-modality intelligent sensory data can address specific quality control tasks, comprehensive evaluation of TCM usually requires the integration of multidimensional information, including form, color, aroma, and taste, which is consistent with the holistic perspective of TCM theory. In this context, multisource information fusion provides a practical way to combine complementary signals from different sensing platforms and to support a more comprehensive assessment of quality [69]. Accordingly, the joint use of electronic nose, electronic tongue, and electronic eye systems has become an important strategy in both food and TCM research. For instance, to address frequent bear bile powder adulteration, ref. [70] employed electronic noses and tongues to capture odor and taste information, respectively. After fusing these datasets and inputting them into an RF model, they achieved 100% classification accuracy. Nevertheless, this near-perfect performance was obtained from a relatively limited dataset of 42 batches and was evaluated mainly through internal dataset splitting rather than independent external validation. In addition, the adulteration setting was restricted to several predefined counterfeit bile powders under controlled experimental conditions, so its robustness across more heterogeneous market samples and analytical platforms remains to be further verified.

Fusion strategies are generally classified into three levels according to the stage at which information is integrated: data-level, feature-level, and decision-level fusion [71,72].

Data-level fusion, also known as low-level fusion, refers to the direct integration of raw signals or minimally preprocessed data from different sensing platforms before feature extraction. Because each modality is retained at an early stage, this strategy can preserve relatively complete sensory information. At the same time, practical implementation is often difficult because multisource data may differ substantially in scale, dimensionality, format, and noise characteristics. Direct alignment can therefore be challenging, and simple concatenation of raw signals may introduce redundancy or amplify noise. For this reason, data-level fusion is still less commonly used in current intelligent sensory studies, although it remains of interest for deep learning frameworks that can learn directly from complex high-dimensional inputs [19,72,73].

Feature-level fusion, also known as intermediate fusion, is currently the most widely used multisensory fusion strategy. In this approach, informative features are first extracted from each modality, such as signal descriptors, image features, or chemically relevant variables. These features are then combined into a joint feature vector for subsequent classification or regression analysis [74]. Compared with data-level fusion, this strategy reduces the difficulty of directly handling heterogeneous raw data while still retaining complementary information from different sensing platforms. Even so, the fused feature space may become highly dimensional, and model performance often depends strongly on feature extraction quality, feature selection, and parameter optimization. A representative example is tea quality grading, where [75] integrated signals from electronic noses, tongues, and vision systems at the feature level. The fused-feature model significantly outperformed single-signal SVM and RF models, achieving 100% accuracy in grade discrimination. Still, this result was obtained from a study-specific dataset consisting of six predefined Longjing tea grades from a single production region, and model evaluation relied mainly on internal train–test splitting and cross-validation rather than independent external validation. Its robustness across broader tea categories, grading standards, and real-market conditions therefore remains to be further verified.

Decision-level fusion, also known as high-level fusion, is performed at the final stage of analysis. In this framework, each sensory modality is modeled independently first, and the resulting outputs are then combined into a unified decision. These outputs may include predicted classes, scores, or probabilities, which can be integrated using strategies such as voting, Bayesian inference, or Dempster–Shafer evidence theory [76]. Compared with data-level and feature-level fusion, this strategy is more flexible because each modality can be analyzed using the most appropriate model. It can also be more tolerant of noise or instability in individual data sources. For example, in the assessment of black tea fermentation, ref. [77] analyzed electrical property data and hyperspectral imaging data separately and then integrated their outputs at the decision level. This approach outperformed single-source models in predicting key quality indicators such as total catechins, soluble sugars, and caffeine, illustrating the practical value of decision-level integration. Even so, the final result depends directly on the reliability of each single-modality model. Insufficient preprocessing, weak feature extraction, or unstable prediction at the individual-model stage may therefore reduce overall fusion performance. This issue is especially relevant in flavor analysis based on electronic noses and tongues, where the number of raw sensor responses may limit the quality of preliminary decisions [78].

Despite the considerable promise of multisource information fusion, its broader application still faces several challenges, including data heterogeneity, information redundancy, and the increasing complexity of feature extraction and model construction. Future work should focus on cross-modal deep learning frameworks that can better capture inter-modal relationships, while also promoting the standardization of data acquisition and preprocessing across batches and laboratories. These advances will be important for translating multisource fusion from controlled experimental studies into more scalable and practical industrial applications [79,80,81,82].

## 4. Data Processing and Analysis Methodologies Powered by Artificial Intelligence

This section summarizes the main artificial intelligence methods used in intelligent sensory evaluation of TCM and MFH products. For clarity, these methods are discussed in two broad categories: traditional machine learning and deep learning. Traditional machine learning, including supervised and unsupervised approaches, is commonly used for classification, regression, and exploratory analysis of sensory data, whereas deep learning is increasingly applied to image analysis, sequential signal modeling, and more complex pattern recognition tasks. The representative algorithms, major analytical tasks, core advantages, main limitations, and typical applications of these methods in TCM/MFH quality evaluation are comparatively summarized in Table 1.

In classical ML frameworks, intelligent sensory data are typically processed through a standard workflow. Feature engineering is first performed, after which the extracted feature vectors are input into the training model. Traditional ML models are broadly classified into supervised and unsupervised learning based on the task objectives. Supervised learning relies on labeled data and aims to establish accurate mapping relationships between input features and output labels. These models are commonly applied in the quality evaluation of TCM and food–medicine dual-use products for identification and classification tasks. For example, in traceability studies of medicinal herb origins, supervised learning models can identify the provenance of unknown samples by integrating chemical fingerprint data from electronic noses and tongues with known origin labels. Additionally, these models can predict processing degree or active ingredient content by learning historical relationships between sensor data acquired during processing and key component concentrations.

Unsupervised learning is effective in analyzing unlabeled data and identifying underlying structures and distributional patterns without prior annotation. In fundamental research on TCM properties and flavors, unsupervised algorithms can automatically identify components with similar structural or sensory features from large and complex chemical datasets. This capability provides important insights into the fundamental relationships between TCM properties, flavors, and their underlying chemical constituents.

**Figure 3:**
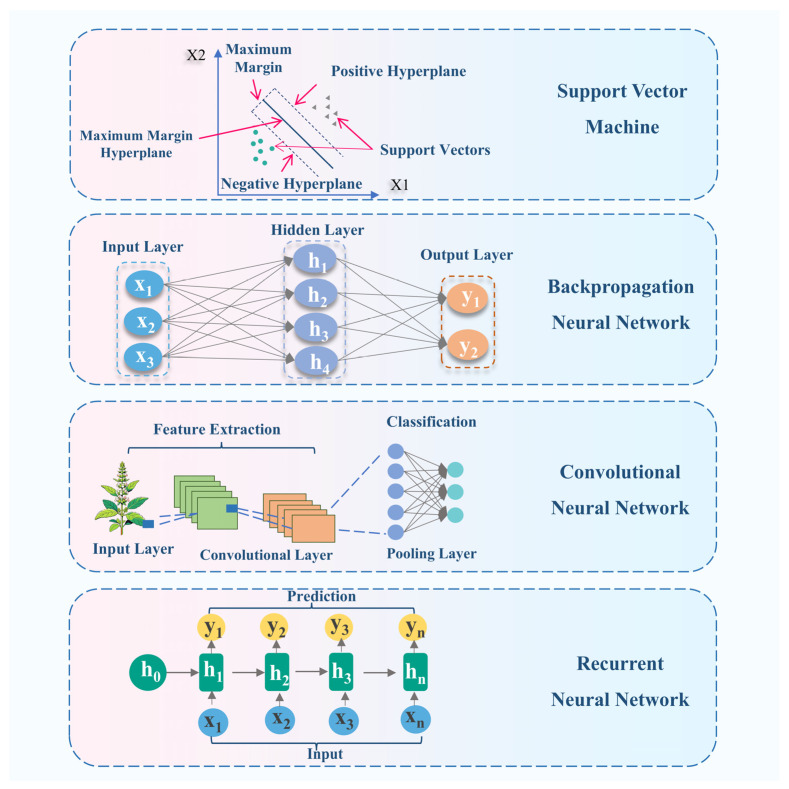
Schematic illustrations of representative machine learning and deep learning algorithms discussed in this review.

DL enables the automated extraction of highly abstract and hierarchical feature representations directly from raw data through neural networks composed of multiple nonlinear layers. This technology is transforming intelligent manufacturing and quality assessment and control in TCM, and its impact continues to expand. For instance, CNNs can be used to analyze high-resolution images of Chinese herbal materials, enabling accurate identification and automated sorting of subtle defects, such as mold or insect damage, and substantially improving quality control efficiency. Moreover, when time-series signals from electronic noses and tongues are treated as one-dimensional sequences and analyzed using LSTM networks, complex relationships between sensor responses and material properties, such as texture or processing temperature, can be revealed. This approach provides data-driven decision support for the intelligent regulation of TCM processing. Figure 3 illustrates schematic representations of common ML algorithms and their application characteristics.

### 4.1. Supervised Learning Algorithms

#### 4.1.1. Support Vector Machine (SVM)

SVM is a classical machine learning method that identifies an optimal separating hyperplane in a high-dimensional feature space and is particularly suitable for classification tasks involving small sample datasets [97]. In intelligent sensory studies of TCM, SVM has been widely used because sensory signals obtained from electronic noses, electronic tongues, and image-based systems are often high-dimensional and require robust discrimination of subtle inter-sample differences. For example, in the geographical origin traceability and quality assessment of medicinal materials, SVM models trained on multidimensional sensory features, such as spectral, texture, and odor information, have enabled rapid and objective identification of commercial herbal products [98]. However, SVM performance may decline when sensor data contain substantial noise or overlapping feature distributions, and its effectiveness often depends on appropriate preprocessing and feature reduction. Therefore, dimensionality reduction methods such as PCA [99,100] or hybrid optimization strategies such as PCA-GWO [101] are frequently introduced to improve classification accuracy and model robustness in practical TCM sensory analysis.

#### 4.1.2. Random Forest (RF)

RF is an ensemble learning method that combines the outputs of multiple decision trees and is widely used for classification and regression tasks in intelligent sensory analysis [32,102]. In TCM studies, RF is particularly useful for high-dimensional sensory datasets because it generally provides robust predictive performance and can also estimate feature importance, thereby helping to identify key variables associated with quality evaluation. In a representative study on the geographical origin traceability and aging-year discrimination of Citri Reticulatae Pericarpium (Chenpi), data from four analytical platforms—GC–MS, GC–IMS, electronic nose, and electronic tongue—were integrated, and RF achieved the best overall performance among seven machine learning models [103]. However, although RF is relatively resistant to overfitting, its interpretability is still lower than that of a single decision tree, and computational cost may increase when a large number of trees are required.

#### 4.1.3. Partial Least Squares Discriminant Analysis (PLS-DA)

PLS-DA is a supervised chemometric method widely used for the classification of high-dimensional sensory and chemical data because it simultaneously performs dimensionality reduction and class discrimination [104]. In intelligent sensory studies of TCM, it is particularly useful for identifying the variables most strongly associated with differences among sample categories. For example, in the processing evaluation of *Aconiti Kusnezoffii* Radix, PLS-DA identified bitterness intensity as the primary taste-related indicator for discriminating different levels of numbing sensation [105]. This analysis further linked the E-tongue bitterness response with sensory scores and the content of diester-diterpenoid alkaloids, thereby providing an objective basis for determining processing endpoints. However, because PLS-DA is essentially a linear method, its performance may be limited when the underlying relationships in the data are strongly nonlinear.

### 4.2. Unsupervised Learning

#### 4.2.1. Principal Component Analysis (PCA)

PCA is a classical unsupervised method widely used for dimensionality reduction and visualization of high-dimensional sensory data [106]. By projecting the original variables onto a smaller number of principal components, PCA can simplify complex datasets, reduce multicollinearity, and reveal the major patterns of variation among samples. In a study on *Coriandrum sativum* L., a high-dimensional flavor profile constructed from GC–MS data and electronic nose responses was analyzed using PCA, which successfully separated 40 coriander varieties in the score plot and visually revealed differences in flavor structure [107]. However, as a linear technique, PCA is limited in its ability to capture complex nonlinear relationships, and the directions of maximum variance are not necessarily the most discriminative for classification tasks.

#### 4.2.2. K-Means Clustering (K-Means)

K-means is a classical unsupervised clustering algorithm that partitions samples into groups according to their similarity to predefined cluster centroids. In intelligent sensory studies, it is mainly used for rapid sample screening and preliminary categorization of unlabeled high-dimensional sensor data. For example, electronic nose data have been analyzed by combining PCA for dimensionality reduction with K-means clustering, achieving effective discrimination among different wine varieties [108]. However, this example also underscores a key limitation of the method: clustering performance depends strongly on the predefined number of clusters (K) and the initial data structure. For chemically complex and categorically unknown TCM samples, determining the optimal K value remains a significant methodological challenge.

### 4.3. Deep Learning Model

#### 4.3.1. Convolutional Neural Network (CNN)

CNNs are a major class of deep learning models that are particularly well suited for image data and other grid-structured sensory signals because they can automatically learn hierarchical feature representations through convolution and pooling operations [109]. In intelligent sensory systems, this capability allows CNNs to capture complex local patterns and spatiotemporal correlations that may be difficult to model using conventional feature-engineering approaches.

To further improve performance, recent studies have incorporated attention mechanisms and structural optimization into CNN-based frameworks. For example, the Adaptive Kernel and Channel Attention network (AKCA-Net) was developed for electronic nose data analysis and achieved 98.21% classification accuracy, with both precision and recall exceeding 98.5%, in soybean geographical origin traceability [110]. In another study on aged Citri Reticulatae Pericarpium, a hybrid 1D CNN–GRU–Attention model integrating digital images and rapid GC-electronic nose data achieved 98.19% accuracy for age discrimination across 0–12-year samples [111]. In addition, SHAP analysis improved model interpretability by identifying key color and texture features associated with classification. Despite these strong results, both models were established on study-specific datasets and evaluated mainly through internal data partitioning rather than independent external validation. For the soybean study, the dataset ultimately derived from a limited number of samples and origins despite repeated measurements, whereas the CRP study relied on samples from a single manufacturer under highly controlled imaging conditions. More broadly, although CNN-based models offer strong predictive performance, they generally require larger datasets and higher computational cost, and their practical robustness still depends on sufficient validation across datasets and platforms.

#### 4.3.2. Long Short-Term Memory (LSTM) Network

LSTM networks are a specialized type of recurrent neural network designed for sequential data analysis and are particularly effective at capturing long-range temporal dependencies [32]. In intelligent sensory systems, LSTMs are well suited for modeling dynamic response signals generated by electronic noses and electronic tongues, where temporal patterns often contain important information for quality evaluation and prediction. For example, in a study on the rapid origin identification of wolfberry, a hybrid LSTM–AM–1DCNN model was developed to analyze fused data from E-nose and E-tongue sensors. On the test set, the model achieved an accuracy of 97.4%, a precision of 97.6%, a recall of 97.4%, and an F1-score of 0.975 [112]. These findings support the value of LSTM-based architectures for temporally structured sensory data. However, their performance still depends on the availability of sufficiently representative sequential datasets and careful model tuning, which may limit their generalizability in small-sample TCM studies.

Overall, no single analytical approach is universally optimal for intelligent sensory evaluation of TCM. Traditional machine learning methods, such as SVM, RF, and PLS-DA, are generally more suitable for small- to medium-sized datasets with manually extracted features, owing to their relatively stable performance and better interpretability. Unsupervised methods, including PCA and K-means, are particularly valuable for exploratory analysis, visualization, and preliminary pattern discovery, although their capacity for high-precision prediction is limited. In contrast, deep learning approaches such as CNNs and LSTMs are more advantageous for image data and complex time-series signals because of their ability to perform automatic feature learning and nonlinear modeling. However, these models usually require larger datasets, higher computational cost, and more rigorous validation to ensure robustness and avoid overfitting. Therefore, the reported performance of different methods should be interpreted in relation to data type, sample size, feature quality, and validation strategy, rather than algorithm complexity alone.

## 5. Specific Applications

The application value of intelligent sensory technologies in TCM quality evaluation is reflected across multiple stages, including raw material authentication, process monitoring, quality grading, and efficacy-related analysis. These application scenarios highlight how odor, taste, appearance, and other sensory dimensions can be incorporated into more structured analytical workflows. At the same time, they also show that intelligent sensory systems are increasingly being used not only for rapid and non-destructive evaluation, but also for linking external sensory traits with internal quality-related information.

**Figure 4:**
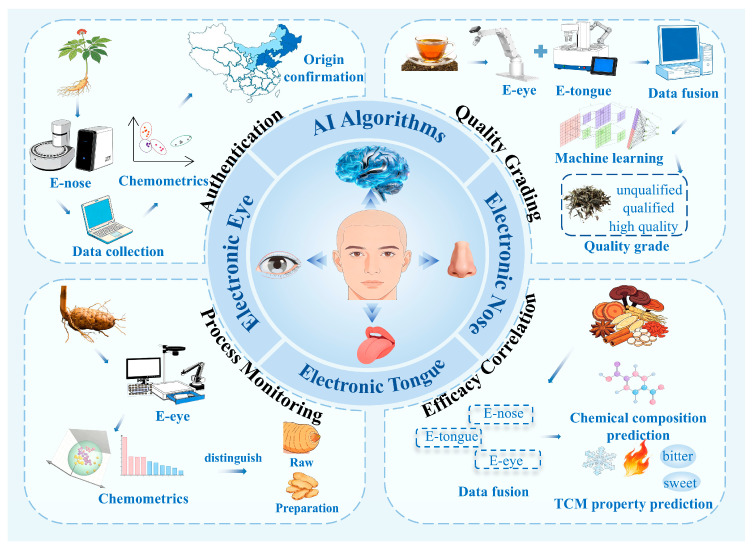
Applications of intelligent sensory technologies integrated with AI algorithms in Traditional Chinese Medicine (TCM) and Medicinal and Food Homology (MFH): raw material identification, processing monitoring, quality classification, and efficacy correlation.

The following sections summarize four representative application scenarios: the authentication of raw materials, monitoring and optimization of herbal processing, quality grading, and the exploration of relationships among sensory attributes, chemical composition, and efficacy. Together, these applications illustrate the practical potential of integrating intelligent sensory systems with AI algorithms for Chinese herbal medicines and medicinal and food homologous products, while also revealing current challenges related to data coverage, cross-platform validation, and consistency with the theoretical framework of TCM. The integration of intelligent sensory systems with AI algorithms for Chinese herbal medicines and food–medicine homologous products is summarized in Figure 4. Recent applications of intelligent sensory technology combined with AI algorithms in TCM research are summarized in Table 2.

### 5.1. Raw Material Authentication: Verification of Origin, Species, and Authenticity

Verification of raw material identity is the first step in ensuring the efficacy and safety of TCM. Traditional identification approaches are often constrained by subjectivity, limited throughput, and insufficient sensitivity to subtle differences caused by geographical origin, botanical variation, or adulteration. Intelligent sensory technologies provide a more objective alternative by capturing multidimensional sensory fingerprints, such as odor, taste, and appearance, and integrating them with artificial intelligence for authentication tasks. In this way, raw material evaluation can move beyond experience-based judgment toward a more standardized and data-driven framework.

In origin and variety identification, methodological development has gradually progressed from conventional chemometric analysis to more integrative and task-adapted models. In *Zanthoxylum bungeanum*, the combined use of electronic nose, electronic tongue, GC-MS, and HPLC data enabled the identification of key flavor compounds associated with geographical authenticity, providing a more mechanistic perspective on regional differentiation [118]. A similar strategy was applied to *Zingiber* species, where RF achieved the best origin classification performance based on GC-MS and rapid GC-E-nose data, while key discriminatory biomarkers such as dimethyl sulfide and α-pinene were simultaneously identified [125]. In turmeric origin tracing, the combination of standard normal variate preprocessing and SVM improved origin discrimination accuracy from 83.3% to 100%, highlighting the importance of preprocessing and algorithm selection for sensory-based authentication [68]. Nevertheless, this result was obtained from a study-specific dataset of 72 batches collected from only three production regions, and model optimization and evaluation were mainly conducted under internal validation settings rather than independent external testing. Its broader robustness across more diverse origins, batches, and real-market conditions therefore remains to be further established.

Recent progress has also included dedicated deep learning models specifically designed for intelligent sensory data. In chili pepper traceability and classification, a sensor-attention convolutional network adaptively weighted multi-channel gas information and achieved nearly 99% accuracy for both variety classification and origin discrimination [61], while feature fusion based on maximum separable discriminant correlation analysis enabled 100% accuracy in chili pepper variety identification [142]. Nevertheless, these strong results were obtained under relatively controlled experimental settings with study-specific datasets, and model evaluation mainly relied on internal train–test splits rather than independent external validation. In addition, the reported datasets were derived from a limited number of varieties or origins with repeated measurements, so their robustness across broader production sources, sensor conditions, and real-market scenarios remains to be further established.

When authentication shifts from natural variation to deliberate adulteration, the analytical focus becomes more target-oriented. In adulterated bear bile powder, E-tongue bitterness and umami responses were strongly correlated with the concentrations of key bioactive components, TUDCA and TCDCA, and an RF model achieved high-precision qualitative and quantitative discrimination [70]. However, this performance was established on a relatively small set of 30 authentic and 12 counterfeit batches, using mainly internal training–test–validation splits under controlled adulteration conditions. Therefore, further validation on independent external samples, broader counterfeit types, and different sensing platforms is still needed before this strategy can be considered broadly transferable for routine market authentication.

Overall, current studies indicate that multisource fusion and task-adapted algorithms generally outperform single-modality or purely descriptive approaches in raw material authentication. At the same time, many reported models are still developed on relatively limited datasets with restricted sample coverage, which may reduce their robustness when applied to broader real-world materials across regions, batches, or cultivation conditions. These findings therefore not only demonstrate the value of intelligent sensory technologies for authentication, but also highlight the need for larger standardized datasets, stronger cross-platform validation, and more generalizable models.

### 5.2. Process Monitoring and Optimization in Herbal Processing

Processing plays a crucial role in shaping the medicinal properties and overall quality of TCM. Traditional assessment of key parameters, such as fire intensity and processing endpoint, often relies on empirical judgment and is therefore difficult to standardize. Intelligent sensory technologies combined with artificial intelligence provide a more objective alternative by tracking multidimensional changes in color, odor, and chemical composition during processing, thereby supporting more quantitative and data-driven process control.

Recent studies have mainly focused on two tasks: real-time process monitoring and parameter optimization. For endpoint determination, E-eye technology combined with FT-NIR spectroscopy was used to monitor surface color changes and the contents of five major bioactive components during the steaming of *Gastrodia elata*. A PLSR model based on FT-NIR data successfully classified the process into raw, partially steamed, and fully steamed stages, enabling real-time stage assessment together with compositional prediction [43]. In the vinegar-processing of *Cyperi Rhizoma*, a deeper multisource fusion strategy integrating computer vision, headspace electronic nose, and HPLC data achieved 100% accuracy in classifying samples according to processing degree using a WOA-optimized RF model [131]. Even so, this result was obtained from a relatively limited dataset of 54 batches and was evaluated mainly through an internal training-test split rather than independent external validation. Notably, perfect classification was achieved only after selecting representative fused features, whereas direct fusion of all experimental data initially yielded much poorer test performance, suggesting that the model’ s robustness may still depend on feature selection strategy and controlled experimental conditions.

Process optimization studies have placed greater emphasis on identifying conditions that preserve characteristic quality attributes. In Guang Chenpi, comparative analysis of different drying methods showed that vacuum freeze-drying best preserved the characteristic volatile profile [130]. In walnut kernel roasting, the integration of electronic nose signals, HS-GC-IMS, and a back-propagation neural network identified 140 °C for 60 min as the optimal roasting condition for maximizing key aroma compounds, thereby providing a quantitative basis for process regulation [129].

Overall, current studies indicate that intelligent sensory technologies can support both dynamic process monitoring and parameter optimization in herbal processing. Multisource fusion is particularly valuable in complex processing scenarios because it captures complementary visual, olfactory, and chemical information. At the same time, most available studies have been conducted under relatively controlled laboratory conditions, and broader industrial application will still require improved sensor robustness, more standardized process datasets, and stronger validation across batches and production environments.

### 5.3. Quality Grading

Quality grading is a key step in safeguarding both the market value and therapeutic efficacy of TCM. Conventional grading practices still rely heavily on manual inspection and experiential judgment, which are often subjective and difficult to standardize. Intelligent sensory technologies combined with artificial intelligence provide a more objective alternative by quantifying visual, olfactory, and other sensory attributes and converting them into reproducible grading criteria.

For general quality evaluation, multisource information fusion has shown clear advantages because it captures complementary sensory information more comprehensively than single-source analysis. In tea quality assessment, the integration of electronic nose, electronic tongue, and computer vision data with machine learning models such as RF achieved 100% accuracy in grade identification [75]. This near-perfect performance, however, was established under a relatively controlled experimental framework involving a single tea type, one production region, and predefined grade categories. Moreover, the model was evaluated mainly by internal dataset splitting and cross-validation, indicating that further validation across independent sample sets, more diverse tea products, and broader commercial grading standards is still needed. A similar pattern was observed in the quality grading of *Ganoderma lucidum* spore powder, where an electronic nose combined with an SVM model reached an accuracy of 98.7% [137]. Even so, the result was derived from a controlled dataset with fixed quality categories from a single production region and was mainly assessed by internal data splitting, while the additional validation set was very limited. Its broader transferability therefore remains to be confirmed.

For medicinal materials in which aging duration is itself a major quality criterion, such as aged tangerine peel (Chenpi), intelligent sensory technologies and AI provide a more refined basis for grading time-dependent physicochemical evolution. In TCM practice, prolonged aging is traditionally associated with superior quality, and this empirical view has increasingly been supported by quantitative evidence [143]. Studies have shown that volatile compounds, such as (+)-limonene and γ-terpinene, together with non-volatile constituents including flavonoids and phenolic acids, undergo systematic transformations during aging. These chemical changes are also accompanied by measurable variations in color and texture. By integrating electronic nose, computer vision, and FT-NIR spectroscopy, models such as RF and CNN–LSTM have achieved aging-year discrimination accuracies ranging from 96.0% to 98.21% [103,111,143,144]. However, these strong results were derived from study-specific sample sets and were assessed mainly by internal calibration-validation partitioning rather than independent external validation. Moreover, while SHAP-based interpretation helped identify key visual features contributing to vintage classification, the quantitative validation of critical volatile markers and the broader robustness of the models across more heterogeneous commercial samples still require further confirmation.

Overall, intelligent sensory technologies combined with AI have substantially improved the objectivity and reproducibility of TCM quality grading. Multisource fusion is particularly advantageous when grading depends on multiple complementary attributes, whereas interpretable models are especially valuable when traditional empirical criteria, such as aging quality, need to be translated into quantitative evidence. However, many current grading models are still developed under relatively narrow sample conditions, and their broader applicability across batches, production regions, and commercial standards remains insufficiently validated. These limitations highlight the importance of larger benchmark datasets, stronger cross-sample validation, and more transparent grading criteria in future development.

### 5.4. Efficacy Correlation and Ingredient Prediction

Beyond raw material authentication, process monitoring, and quality grading, an emerging application of intelligent sensory technologies combined with AI is to explore links among sensory attributes, chemical composition, and functional efficacy in TCM. Current studies in this area can be broadly divided into two directions: rapid prediction of key chemical components and bio-inspired screening aimed at connecting sensory perception with bioactive constituents and pharmacological effects.

For rapid component prediction, intelligent sensory technologies provide a non-destructive and efficient alternative to conventional chemical analysis by establishing quantitative relationships between rapid physical or spectral signals and specific compositional parameters. For example, in date syrup quality assessment, hyperspectral imaging combined with artificial neural network models enabled the accurate prediction of key quality parameters, including sucrose, proline, ash content, and the fructose-to-glucose ratio, thereby illustrating the potential of intelligent sensory systems for real-time compositional evaluation [50].

A more mechanistically informative direction involves receptor-guided or biomimetic screening. Unlike conventional electronic tongue systems that mainly depend on array-response pattern recognition, biosensor-based platforms can identify active compounds through specific receptor interactions, thereby providing a more interpretable link between sensory properties and efficacy-related substances. In Huangqi Shengmai Yin, a biosensor based on the sweet taste receptor TAS1R2/3 identified five key sweetness-related quality markers, including calycosin. Subsequent validation in a zebrafish model further showed that these compounds could mitigate vascular injury and enhance immune function, thereby establishing a preliminary evidence chain from sweetness perception to chemical constituents and therapeutic activity [138]. Similarly, in Huangjing Zanyu Capsules, a biosensor based on the pungent receptor TRPV1 identified schisandrin A, and pharmacological experiments further confirmed its efficacy in improving oligoasthenospermia through the regulation of autophagy and apoptosis in testicular tissue [139]. These studies illustrate a methodological progression from sensory-associated correlation toward receptor-guided bioactive constituent discovery and functional verification.

A related extension is the use of multisource intelligent sensory data to support the objective characterization of holistic medicinal properties, including the four natures and five flavors of TCM [141,145]. Although this direction remains more exploratory than direct component prediction, it expands the role of intelligent sensory technologies from analytical identification toward theory-oriented interpretation of TCM properties.

Overall, current studies suggest that ingredient prediction is closer to immediate quality-control application because it directly supports rapid and non-destructive estimation of measurable chemical parameters. By contrast, efficacy-oriented correlation and receptor-guided screening offer greater theoretical value by helping bridge sensory attributes, active constituents, and pharmacological functions. Even so, these latter approaches remain at a relatively early stage and still require stronger validation, clearer mechanistic interpretation, and deeper integration with the theoretical framework of TCM.

## 6. Current Challenges and Future Outlook

### 6.1. Current Challenges

The integration of intelligent sensory technologies with artificial intelligence is reshaping the paradigm of quality assessment for TCM and MFH products. To translate this interdisciplinary field from innovative laboratory research into large-scale industrial application, several critical challenges must be addressed. These include the establishment of robust and standardized data foundations, the improvement of model robustness and generalizability, and the effective integration of technological advances with the theoretical framework of traditional TCM.

#### 6.1.1. Data Bottlenecks: Insufficient Quality, Standardization, and Sharing Mechanisms

High-quality data remain the primary constraint on the broader application of artificial intelligence in intelligent sensory evaluation. In this field, datasets are often limited not only in size, but also in representativeness and comparability. Small-sample problems are particularly prominent for genuine medicinal materials with restricted geographical origins, as well as for aged products such as Chenpi, which require long production cycles before stable quality characteristics can be established. As a result, many studies rely on narrowly distributed sample sets, which restricts model robustness and weakens external validity [146,147].

A second bottleneck is the scarcity of reliable annotations. Data labeling in TCM sensory research still depends heavily on expert judgment, sensory evaluation, or experience-based categorization, which is labor-intensive and vulnerable to subjective inconsistency. This limits the availability of large-scale, high-quality annotated datasets and makes it difficult to support robust training and validation, particularly for data-hungry deep learning models [148].

Standardization is another major weakness. Differences in sensor platforms, sampling procedures, environmental conditions, and preprocessing workflows across laboratories can introduce substantial batch effects and reduce inter-study comparability. Recent reviews have emphasized that sensor drift compensation, calibration reliability, and sensor standardization remain major obstacles to the broader practical deployment of E-nose and E-tongue technologies. Without unified standards for data acquisition, annotation, and reporting, datasets remain fragmented and difficult to reuse, which greatly limits data sharing and cumulative model development [149,150,151].

These problems are further amplified in multisource studies. Although multimodal fusion is expected to provide a more holistic characterization of TCM quality, effective integration is often hindered by mismatched data structures, inconsistent scales, and incomplete cross-modal alignment. Therefore, the current bottleneck is not merely the lack of data volume, but the absence of well-curated, standardized, and interoperable datasets that can support reproducible and scalable intelligent sensory analysis [152].

#### 6.1.2. Model Bottlenecks: Insufficient Generalizability, Interpretability, and Robustness

At the model level, one of the main barriers to practical deployment is insufficient generalizability. Models that perform well on controlled experimental datasets often exhibit marked performance declines when applied to real-world samples that vary in geographical origin, production batch, cultivation conditions, or processing status. This suggests that many current models remain overly dependent on specific training conditions and do not adapt well to the natural variability of TCM materials [146].

A second bottleneck is limited interpretability. Many deep learning models achieve high predictive performance, but their decisions making processes remain difficult to explain in mechanistic or domain-relevant terms. In the context of TCM quality evaluation, this lack of transparency weakens the confidence of regulators, manufacturers, and end-users and reduces the practical acceptability of these models in quality control scenarios that require traceable and scientifically interpretable decisions [148].

Model robustness is also strongly affected by sensor drift and environmental variation. Intelligent sensory devices, particularly electronic noses and electronic tongues, are susceptible to baseline drift during prolonged operation or under changing temperature and humidity conditions. As a result, models trained on initially calibrated data may gradually lose predictive accuracy after deployment. This highlights a key practical limitation: many current algorithms are not sufficiently robust to maintain stable performance under dynamic real-world conditions, making recalibration and model updating both necessary and resource-intensive [149,153].

#### 6.1.3. Bottlenecks in Integrating Technology and Theory: Bridging the Gap from Correlation to Mechanism

A major bottleneck in the current development of intelligent sensory evaluation is the insufficient integration of data-driven technologies with the theoretical framework of TCM. Most existing studies remain at the level of correlation analysis, for example by using artificial intelligence to classify samples or predict sensory categories, while paying much less attention to how sensory fingerprints relate to medicinal properties, flavors, channel tropism, and therapeutic action in a mechanistically interpretable way. As a result, many current models can improve predictive performance, but still provide limited support for explaining the material basis and theoretical connotation of TCM quality attributes [148,154].

For intelligent sensory systems to contribute to a modern and interpretable framework for herbal medicine, TCM knowledge needs to be incorporated more deeply into model design rather than being treated merely as a superficial label or annotation. This requires moving beyond simple pattern recognition toward integrative frameworks that combine sensory signals, chemical composition, pharmacological evidence, and TCM theory in a coordinated manner [154,155].

A related practical challenge is that sensing equipment specifically adapted to the complex matrices and application scenarios of TCM is still underdeveloped. Current devices are often constrained by portability, cost, and long-term robustness, especially under field or industrial conditions. These limitations restrict their wider deployment in cultivation, harvesting, processing, and on-site quality control, and therefore slow the translation of intelligent sensory technologies from laboratory demonstration to practical use [146,156].

### 6.2. Future Outlook

To address the current challenges, future advancements should focus on systematically overcoming bottlenecks in data, models, and theory integration through technological innovation and interdisciplinary collaboration. Efforts should also be directed toward exploring new application paradigms, thereby advancing the digitalization and intelligence of the quality evaluation system for TCM.

#### 6.2.1. Building Robust Data Infrastructure and Adaptive Modeling Strategies

To address the persistent problem of limited sample size, more flexible learning strategies will be needed. Few-shot and transfer learning may provide useful solutions by adapting feature representations learned from larger general-purpose datasets to specific TCM sensory tasks, thereby improving model performance under data-constrained conditions [157]. In addition, GAN-based frameworks may offer a potential strategy for data augmentation, although the reliability, representativeness, and safety of synthetic sensory data still require rigorous validation before routine use in TCM quality evaluation. Self-supervised learning also shows promise for extracting informative feature representations from large volumes of unlabeled data, which could reduce dependence on labor-intensive manual annotation [158,159].

To overcome data silos, the establishment of a standardized and interoperable industry-wide database remains essential. This will require coordinated collaboration among academia, industry, and regulatory bodies to develop standardized operating procedures, annotation criteria, and benchmark datasets. At the same time, future research should focus on developing more adaptive models capable of coping with sensor drift and environmental variation. By incorporating continuous learning or recalibration mechanisms, such models may improve long-term stability in real-world deployments, although their robustness across batches, platforms, and laboratories still needs systematic validation.

#### 6.2.2. Advancing Explainable and Deployable Intelligent Sensory Systems

To improve model credibility and practical acceptability, greater emphasis should be placed on explainable artificial intelligence. Interpretability tools such as SHAP and LIME may help clarify the contribution of specific sensory features to model outputs, while attention-based or other inherently more interpretable architectures could improve transparency in domain-oriented decision making [160]. In addition, future research may benefit from human–machine collaborative frameworks that combine the high-throughput analytical capacity of AI with expert knowledge and causal reasoning in TCM. Such hybrid systems could support more reliable and context-aware decision making, although their practical implementation still requires careful workflow design and validation [159].

At the hardware level, further progress in intelligent sensory evaluation will depend on the development of more portable, cost-effective, and robust sensing devices. Advances in nanomaterials and biomimetic design may improve sensor selectivity and long-term stability, but their practical value must still be verified under realistic industrial and field conditions. At the same time, miniaturization and system integration will be important for expanding accessibility beyond laboratory settings. In the longer term, the integration of sensing devices with Internet of Things (IoT) infrastructure may support more continuous data acquisition across cultivation, processing, and distribution stages, provided that challenges related to calibration, standardization, and data interoperability can be adequately addressed [27].

#### 6.2.3. Promoting Mechanism-Oriented Integration and Future Value Creation

Future research should move beyond conventional identification and grading toward a more systematic exploration of the relationships among sensory attributes, chemical composition, and functional efficacy. By integrating intelligent sensory fingerprints with multi-omics data and biomimetic sensing strategies, it may become possible to identify quality markers that link specific sensory profiles with defined biological activities. Such efforts could help provide a more evidence-based interpretation of traditional pharmacological concepts, including medicinal properties, flavors, and channel tropism [154,155].

In the field of medicinal and food homology (MFH), artificial intelligence may also support the future development of more personalized health products. For example, the integration of individual health information with sensory and compositional profiles could assist in the design of more targeted herbal formulations or functional ingredient combinations. However, such applications remain dependent on reliable multimodal datasets, validated efficacy markers, and appropriate regulatory frameworks.

In the longer term, intelligent sensory technologies may contribute to a more digital and traceable TCM industry chain. The deployment of distributed sensing nodes combined with centralized data analytics could support more continuous monitoring, improved decision making, and stronger traceability from raw materials to finished products. Nevertheless, the realization of such an integrated ecosystem will still depend on substantial progress in standardization, device robustness, cost control, and cross-platform data interoperability [146,156].

## 7. Conclusions

The integration of intelligent sensory technologies with artificial intelligence is reshaping the quality evaluation of traditional Chinese medicine (TCM) from subjective sensory judgment toward a more objective, data-driven, and system-oriented framework. This review systematically summarizes the basic principles and applications of key intelligent sensory systems, including the electronic nose, electronic tongue, and electronic eye, and highlights how artificial intelligence methods support signal processing, pattern recognition, classification, regression, and multisource information fusion in TCM quality assessment. Collectively, these technologies provide new possibilities for the authentication, grading, processing evaluation, and efficacy-related interpretation of TCM and medicinal and food homologous products.

At the same time, this review also indicates that current progress is still constrained by several major challenges, including limited data quality and standardization, insufficient model generalizability and interpretability, and the incomplete integration of intelligent sensing results with the theoretical framework of TCM. Therefore, the main contribution of artificial intelligence and intelligent sensory technologies lies not only in improving analytical efficiency and objectivity, but also in creating the foundation for a more holistic and mechanism-oriented understanding of TCM quality.

Future research should focus on the construction of standardized and sharable datasets, the development of robust and explainable models, and the deeper integration of sensory fingerprints with chemical, pharmacological, and theoretical evidence. Continued progress in these directions will be essential for promoting the practical deployment, scientific modernization, and broader international acceptance of TCM quality evaluation.

## Figures and Tables

**Table 1:** Comparative summary of representative statistical and artificial intelligence methods used in intelligent sensory evaluation of TCM and MFH.

Algorithm Type	Representative Algorithms	Main Analytical Task	Core Advantages	Main Limitations	Typical Applications in TCM/MFH	Refs.
Traditional ML	Linear Regression/Logistic Regression	Regression; classification	Simple model structure and strong interpretability	Assumes linear relationships and has limited ability to capture complex patterns	Quality standard research of TCM	[83]
	SVM	Classification; regression	Suitable for small-sample and high-dimensional data; solid theoretical foundation	Sensitive to parameter and kernel function selection, with slow training on large-scale data	Comprehensive Quality Evaluation of TCM	[84,85]
	Decision Tree	Classification	Intuitive and easy to interpret	Prone to overfitting; sensitive to data fluctuations	Quantification of Medicinal Properties of TCM	[86]
	RF	Classification; regression; feature importance analysis	Handles high-dimensional data well; resistant to overfitting; can assess feature importance	Limited interpretability; computational cost may increase with model complexity	Geographical origin traceability	[87]
	XGBoost	Classification; regression	High prediction accuracy, effectively handles missing values and complex nonlinear relationships	Many parameters; optimization can be complex	Ecological quality assessment of TCM	[88]
	K-means	Clustering	Simple algorithm with high computational efficiency	Requires predefinition of cluster number; sensitive to noise and outliers	Authenticity identification of TCM	[89]
	PCA	Dimensionality reduction; visualization	Intuitive visualization; reduces multicollinearity; no labels required	Captures only linear relationships	Food adulteration identification	[90]
Deep Learning	Self-Supervised Learning	Representation learning from unlabeled data	Reduces dependence on expensive labeled data; useful for large unlabeled datasets	Pretext task design remains challenging	Potential identification of new TCM varieties and large-scale sensory data mining	[91]
	CNN	Feature extraction; classification	Strong automatic spatial feature learning; avoids manual feature design	Requires large labeled datasets and high computational resources	Identification of medicinal plant varieties; image-based quality evaluation	[92]
	RNN/LSTM	Sequential modeling; classification; prediction	Suitable for dynamic processes and sequential data; captures temporal dependencies	Training and parameter tuning can be complex	Adulteration identification; dynamic sensory signal analysis	[93]
	GNN	Relational learning; graph-based analysis	Capable of modeling non-Euclidean data and complex topological relationships	Computationally complex; depends on graph construction quality	Potential constituent–target–efficacy relationship modeling	[94]
	GAN	Data augmentation; synthetic data generation	Generates realistic data and can alleviate data scarcity	Training instability and risk of mode collapse	Potential data augmentation for limited sensory datasets	[95]
	Autoencoder	Nonlinear dimensionality reduction; denoising; feature extraction	Effective for data compression, denoising, and latent feature learning	Learned representations may not align with downstream tasks	Potential latent feature learning	[96]

**Table 2:** Representative applications of intelligent sensory instruments and data analysis methods in TCM and MFH.

Application Task	Sample/Material	Sensory Platform	Algorithm Type/Model	Main Result/Performance	Refs.
Origin Traceability	*Angelica dahurica* samples	E-nose	Deep learning/BM-Net	BM-Net enabled high-accuracy origin discrimination, achieving 97.75% accuracy for wide-range origins and 96.08% accuracy for small-range origins, with consistently high precision and recall	[113]
	Soybean samples	E-nose	Deep learning/AKCA-Net	AKCA-Net achieved superior origin traceability performance, with 98.21% accuracy, 98.57% precision, and 98.60% recall	[110]
	*Codonopsis Radix* samples	E-nose, E-tongue	Supervised learning/PLS-DA	Multisource fusion of E-nose and E-tongue data improved origin identification; the PLS-DA model on z-score normalized fused data provided the most balanced discrimination performance	[114]
	Wolfberry fruit samples	Vis-NIR HSI	Deep learning/S-IFCNN	The S-IFCNN model effectively fused spectral and image features for geographical origin identification, achieving 91.99% accuracy	[115]
	Citri Reticulatae Pericarpium (Guang Chenpi) samples	GC-MS, GC-IMS, E-nose, E-tongue	Supervised learning/RF, PLS-DA	Aging affected flavor more strongly than origin; the RF model achieved 100% accuracy for origin discrimination and 96% accuracy for aging year prediction	[103]
	Chenpi samples	Computer Vision, UF-GC-E-nose	Deep learning/BPNN	Fusion of computer vision and UF-GC-E-nose data identified 57 discriminative marker traits and achieved 100% accuracy in origin discrimination	[116]
	*Zanthoxylum bungeanum* samples	HS-SPME-GC-MS, E-nose	Unsupervised learning/PCA	E-nose combined with GC-MS enabled preliminary discrimination of huajiao from different origins and varieties, while key terpenoid biomarkers supported regional and cultivar differentiation	[117]
	*Zanthoxylum bungeanum* samples	E-nose, E-tongue, GC-MS, HPLC	Chemometric analysis/PCA-entropy model	Multidimensional analysis combined with PCA-entropy modeling revealed significant climate–quality relationships, providing an objective basis for regional quality differentiation	[118]
	*Ocinum × citriodorum* samples	E-nose, E-tongue, HS-GC-IMS, HS-SPME-GC-MS	Supervised learning/OPLS-DA	A dual-modality sensory–chemical framework identified 33 origin-discriminatory VOC markers and effectively differentiated samples from distinct geographical regions	[119]
	Chili pepper samples	E-nose	Deep learning/SACNet	SACNet showed excellent performance in variety classification and origin traceability, achieving 98.56%, 97.43%, and 99.31% accuracy across different datasets	[61]
	Frankincense samples	E-nose, HS-SPME-GC-MS	Supervised learning/PLS-DA	PLS-DA effectively distinguished frankincense from Oman/Somalia and other origins; 149 VOCs were characterized, and p-cymenol was identified as a major contributor to citrus aroma	[120]
	Chinese jujube fruit samples	Computer Vision, UF-GC-E-nose, GC-MS	Supervised learning/SVM	Multidimensional feature fusion identified 46 discriminative trait markers and achieved 100.0% accuracy in origin discrimination using the optimized SVM model	[98]
Variety differentiation	*Polygonati Rhizoma* and *Polygonati Odorati Rhizoma* samples	HS-GC-IMS, E-nose	Supervised learning/OPLS-DA	Combined E-nose and GC-IMS analysis identified 16 key differential VOCs and enabled effective discrimination between PR and POR samples	[121]
	*Alpinia galanga* and *Myristica fragrans* samples	E-nose, HS-GC-MS, UPLC-QTOF-MS	Chemometric analysis/PCA, OPLS-DA	Integrated volatile and non-volatile metabolite analysis identified key discriminatory compounds and demonstrated superior antioxidant capacity in Myristica fragrans	[122]
	Jujube fruit samples	HSI (VNIR + SWIR), 2DCOS-CARS	Deep learning/PSO-CNN-BiGRU	The PSO-CNN-BiGRU model based on fused VNIR and SWIR hyperspectral data achieved 98.26% accuracy, 98.41% precision, 99.75% specificity, and 98.26% sensitivity	[123]
	*Gentiana macrophylla* samples	E-eye, E-nose, HS-SPME-GC-MS, HPLC	Chemometric analysis/PCA, OPLS-DA	Fusion of intelligent sensory technologies with chemical analysis enabled rapid discrimination between wild and cultivated samples, with distinct volatile and compositional markers identified	[124]
	Coriander samples	HS-SPME, GC-MS, E-nose	Unsupervised learning/HCA, PCA	A total of 207 volatile compounds and 37 aroma-active components were identified; HCA and PCA effectively differentiated 40 coriander varieties	[107]
	Ginger rhizome samples	HS-GC-MS, Fast GC E-nose	Supervised learning/RF	HS-GC-MS and fast GC E-nose enabled rapid discrimination of ginger varieties and geographical origins, with RF showing the highest classification accuracy among compared models	[125]
Authenticity verification	Bear bile powder samples	E-tongue, E-nose, GC-MS	Supervised learning/RF	RF achieved the best qualitative and quantitative performance, with 100% accuracy, precision, recall, and F1-score for authentication, as well as the highest R^2^ and lowest RMSE for content prediction	[70]
	Honey samples	E-tongue, E-eye	Supervised learning/SVR	Computer vision enabled highly accurate adulteration prediction, with RMSE = 0.46% and R^2^ = 0.9993; voltammetric E-tongue showed even higher predictive accuracy (RMSE = 0.25%, R^2^ = 0.9998)	[126]
	Red chili powder samples	E-eye	Deep learning/1D-CNN, 2D-CNN	CNN-based computer vision models demonstrated feasibility for adulteration detection, with 1D-CNN and 2D-CNN achieving test accuracies of 84.56% and 84.62%, respectively	[127]
Processing and preparation	*Aurantii Fructus* samples	SEM, Ultrafast GC E-nose	Unsupervised learning/PCA	Drying at 55 °C provided the best balance between product quality and aroma preservation; PCA revealed significant changes in volatile profiles during the drying process	[128]
	Walnut kernel samples	E-nose, HS-SPME-GC-MS, HS-GC-IMS	Deep learning/BP Neural Network	Roasting at 140 °C for 60 min was identified as the optimal condition for aroma enhancement, and the backpropagation neural network predicted VOC contents with satisfactory accuracy (0.9448)	[129]
	*Guang Chenpi* samples	E-nose, GC-IMS, HS-SPME-GC-MS	Chemometric analysis/PCA, OPLS-DA	Vacuum-freeze drying preserved the highest VOC content and the richest volatile composition, and chemometric analysis identified terpenes and esters as the main differential metabolites	[130]
	*Cyperus Rhizome* samples	E-eye, Flash GC, E-nose, HPLC	Supervised learning/WOA-RF	Multisource fusion combined with the WOA-RF model achieved 100% classification accuracy for vinegar-processed samples at different roasting levels	[131]
	*Gardeniae Fructus* samples	E-eye, HPLC, E-nose, HS-SPME-GC-MS, GC-IMS	Chemometric analysis/HCA, PLS-DA	Integrated sensory and chemical analysis identified 28 key flavor compounds and clarified that the burnt aroma mainly originated from Maillard/caramelization reactions and lipid oxidation during stir-frying	[132]
	*Gardeniae Fructus* samples	HPLC, UHPLC-Q-TOF-MS, Ultrafast GC E-nose	Chemometric analysis/PCA, OPLS-DA	Identified the differential chemical components in *Gardeniae Fructus* before and after ginger juice processing, and pinpointed multiple specific non-volatile and volatile markers	[133]
	*Mentha spicata* L. samples	E-nose, GC-MS	Supervised learning/Nu-SVM	Hot-air drying produced the highest essential oil yield, and the Nu-SVM model classified eight essential oil groups with 97.5% accuracy	[134]
	*Moutan Cortex* samples	E-nose, HPLC	Supervised learning/PLSR, SVR	Achieved objective identification between raw and carbonized *Moutan Cortex*, and established an odor-based chemical content prediction model	[135]
	*Psoralea corylifolia* fructus samples	E-eye, E-nose, HPLC	Chemometric analysis/PCA, OPLS-DA	Established an innovative method for rapid discrimination between raw and salt-processed *Psoralea corylifolia* fructus by integrating multisource sensory and analytical data	[136]
Quality grading	Chenpi samples	Computer Vision, Flash GC E-nose	Deep learning/1D-CNN-GRU-Attention, SHAP	The 1D-CNN-GRU-Attention model achieved 98.19% classification accuracy for aging year discrimination, while SHAP analysis improved interpretability by identifying key color and texture features	[111]
	Tea samples	E-nose, E-tongue, E-eye	Supervised learning/RF, SVM, PLS	100% accurate tea grade identification via feature-level fusion of multisource information; fused signals showed superior performance in quantitative chemical prediction	[75]
	*Ganoderma lucidum* spore powder samples	E-nose, FTIR, UV-Vis	Supervised learning/MeanSVM	MeanSVM provided the highest quality classification accuracy (98.7%), and the E-nose-based method achieved 100% correct classification for validation samples	[137]
	*Atractylodes macrocephala*	E-nose, HPLC	Supervised learning/XGBoost	Machine learning and SHAP analysis identified aroma-related features and atractylon as key markers for distinguishing high-quality samples	[36]
Efficacy correlation	Huangqi Shengmai Yin samples	Biosensor, UPLC-MS	Biosensing strategy/receptor-based screening	Sweet-taste receptor biosensor screened 5 strongly binding components with proven efficacy in improving vascular damage and enhancing immunity	[138]
	Huangjing Zanyu Capsules	Biosensor	Biosensing strategy/receptor-based screening	Revealed that schisandrin A improves oligoasthenospermia by effectively binding to c-kit, antagonizing TRPV1 to inhibit autophagy, and ultimately reversing apoptosis	[139]
	aged tangerine peel	GC-IMS, GC-MS, E-nose, Molecular Docking	Chemometric analysis/PCA	Clarified aging-related aroma evolution in aged tangerine peel; revealed molecular basis for depression prevention by 10 key aroma compounds	[140]
	97 types of TCM decoction pieces	E-nose, E-tongue	Supervised learning/PLS-DA, LS-SVM	Developed a prediction model for the cold/hot nature of TCM based on multisource electronic sensory information, achieving a correct classification rate of 85.57%	[141]

## Data Availability

No new data were created or analyzed in this study.
